# A New Dressing in Surgery

**Published:** 1896-06

**Authors:** 


### A New Dressing in Surgery.

Dr. Welch, the bacteriologist of the Johns Hopkins Hospital, has demonstrated that germs will not grow in the immediate vicinity of silver. His discovery is made use of in the dressing of aseptic surgical wounds, by placing silver foil immediately in contact with the closed incision in sheets about four inches square. The other aseptic dressings are then applied.—*Medical Times.*

